# Multimodal Imaging and Biomarkers in Cardiac Amyloidosis

**DOI:** 10.3390/diagnostics12030627

**Published:** 2022-03-03

**Authors:** Mi-Hyang Jung, Suyon Chang, Eun Ji Han, Jong-Chan Youn

**Affiliations:** 1 Division of Cardiology, Department of Internal Medicine, Seoul St. Mary’s Hospital, College of Medicine, The Catholic University of Korea, Seoul 06591, Korea; floria0515@gmail.com; 2 Department of Radiology, Seoul St. Mary’s Hospital, College of Medicine, The Catholic University of Korea, Seoul 06591, Korea; ohyes723@gmail.com; 3 Division of Nuclear Medicine, Department of Radiology, Yeouido St. Mary’s Hospital, College of Medicine, The Catholic University of Korea, Seoul 06591, Korea; iwao@catholic.ac.kr; 4 Catholic Research Institute for Intractable Cardiovascular Disease, College of Medicine, The Catholic University of Korea, Seoul 06591, Korea

**Keywords:** amyloidosis, immunoglobulin light chain amyloidosis, transthyretin amyloidosis, multimodal imaging, biomarkers

## Abstract

Amyloidosis is a progressive infiltrative disease instigated by the extracellular deposition of amyloid fibrils in various organs such as the heart, kidney, and peripheral nerves. Cardiac amyloid deposits cause restrictive cardiomyopathy, leading to a poor prognosis in systemic amyloidosis. The most common etiologies of cardiac amyloidosis (CA) are immunoglobulin light chain deposits (AL-CA) and misfolded transthyretin deposits (ATTR-CA). In recent years, many developments have been accomplished in the field of diagnosis and treatment of CA. At present, ATTR-CA can be noninvasively diagnosed if the following two conditions are fulfilled in the setting of typical echocardiographic/cardiac MRI findings: (1) grade 2 or 3 myocardial uptake in bone scintigraphy confirmed by SPECT and (2) absence of monoclonal protein confirmed by serum-free light chain assay, and serum/urine protein electrophoresis with immunofixation test. Effective therapies are evolving in both types of CA (tafamidis for ATTR-CA and immunologic treatments for AL-CA). Thus, early suspicion and prompt diagnosis are crucial for achieving better outcomes. In this review, we have summarized the role of multimodal imaging (e.g., echocardiography, cardiac MRI, and bone scintigraphy) and biomarkers (e.g., troponin, BNP) in the diagnosis, risk stratification, and treatment monitoring of CA.

## 1. Introduction

Systemic amyloidosis refers to a progressive infiltrative disease attributed to the extracellular deposition of abnormal proteins that aggregate and form amyloid fibrils in diverse organs. Cardiac amyloid deposits result in restrictive cardiomyopathy and are concomitant with poor prognosis in systemic amyloidosis [1,2,3]. Although cardiac amyloidosis (CA) is an underrecognized cause of heart failure, recent advances in multimodal cardiac imaging combined with innovative therapeutic agents have facilitated earlier diagnosis and improved clinical outcomes [4,5,6,7,8,9,10]. Thus, early suspicion and timely diagnosis using multimodal imaging and biomarkers is of paramount importance in the management of CA as the early-stage disease is more effectively manageable with newer therapeutic modalities. Unfortunately, the overall prognosis is dismal in untreated CA patients [8,10].

## 2. Overview of CA

**Figure 1:**
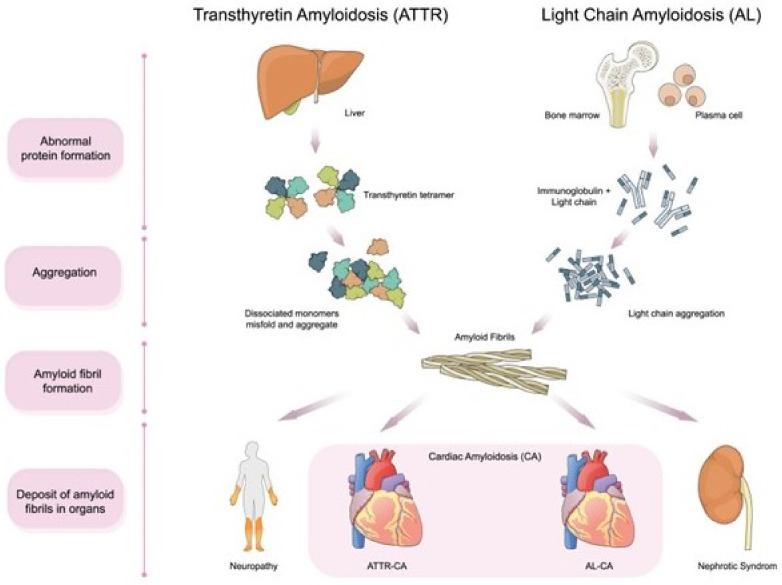
Overview of cardiac amyloidosis.

Among more than 30 known amyloidogenic proteins, immunoglobulin light chain (AL) and transthyretin (ATTR) are the two predominant CA-associated proteins. They have been often found to infiltrate the heart and result in restrictive cardiomyopathy (Figure 1). Accordingly, the AL-CA is resultant due to the deposition of immunoglobulin light chains, which are produced by abnormal plasma cells. While the development of ATTR-CA is attributed to the deposition of misfolded transport protein for thyroid hormone and retinol—transthyretin (TTR)—which is primarily produced by the liver. Depending on the presence or absence of mutation in the ATTR gene, ATTR-CA is further classified into either wild-type ATTR-CA (ATTRwt, mutation absent, >90% of cases) or hereditary ATTR-CA (ATTRv, v for variant, mutation present, <10% of cases), respectively. After confirmation of ATTR, genetic testing for TTR mutation should be considered. Through genetic testing, we can differentiate between ATTRwt and ATTRv and further identify the family members who might need genetic counseling. The kidney (leading to nephrotic syndrome) followed by the heart are the top two prevalent sites of AL involvement, occurring in 50–75% of all AL patients. In the case of ATTR, heart involvement is found in almost all patients with ATTRwt, while some patients with ATTRv do not show heart involvement [4,5]. For instance, the Val30Met mutation causes predominantly neuropathy, also known as familial amyloidotic polyneuropathy (FAP). A previous study involving patients with Val30Met FAP showed differential cardiac involvement based on the type of amyloid fibrils [11]. Although both AL- and ATTR-CA result in restrictive cardiomyopathy, AL-CA exhibits a quicker progressive course, with the median survival being less than six months in untreated cases. This difference in clinical progression of the two CA manifestations implies that AL-CA possesses features of both toxic and infiltrative cardiomyopathy [2,12,13].

## 3. Clinical Presentation

The clinical presentation of CA involves symptoms and signs of heart failure such as dyspnea on exertion and lower leg edema. Moreover, relatively low blood pressure due to low cardiac output or intolerance to blood pressure-lowering drugs further raises the possibility of CA. Unfortunately, the diagnosis of CA remains challenging due to several factors. These include physician-related factors such as nonfamiliarity with new diagnostic algorithm, confusion with AL- and ATTR-CA, clinical nihilism that originates from the past knowledge that there are few effective therapies in CA, and disease-related factors such as the relative rarity of the disease, nonspecific symptoms, and clinical overlap with other diseases that cause left ventricular hypertrophy. Indeed, ATTRwt-CA is reported to be found in approximately 17% of heart failure with preserved ejection fraction cases [14,15], ~16% of severe aortic stenosis cases (particularly for low-flow, low-gradient severe aortic stenosis) [16,17], and ~5% of hypertrophic cardiomyopathy cases who were subjected to myectomy [18,19]. Although left ventricular ejection fraction is usually preserved in CA, it can also be manifested across the entire spectrum of left ventricular ejection fraction [20]. In addition to heart failure, CA is often accompanied by diseases of the conduction system and arrhythmia [21]. Atrial fibrillation often displays a controlled ventricular response due to underlying conduction disease and is persistent in most CA cases. Patients may develop fatal conduction disturbances such as atrioventricular block or symptomatic bradycardia, which necessitate pacemaker implantation. In selected cases with history of ventricular arrhythmia, implantable cardioverter-defibrillators (ICD) should be considered. Additionally, the risk of intracardiac thrombus may occur even in sinus rhythm, possibly due to atrial involvement of the amyloid deposit [22].

Extracardiac symptoms, such as carpal tunnel syndrome, spinal stenosis, spontaneous biceps tendon rupture, autonomic dysfunction, nephrotic syndrome (particularly for AL amyloidosis), macroglossia, and skin bruising, have also been documented to precede CA [1,5,23,24,25]. These symptoms are termed as “red flag” signs of CA. Thus, unexplained increased left ventricular wall thickness (≥12 mm) combined with any of the “red flag” signs of CA should be promptly considered for further evaluation [8].

## 4. Diagnosis and Treatment Monitoring

### 4.1. Electrocardiography

Although nonspecific, electrocardiography (ECG) serves an important role in raising clinical suspicion for CA manifestation. The hallmark feature of ECG in the case of CA is a disproportionately low QRS voltage to left ventricular mass (QRS amplitude < 5 mm in the limb leads or <10 mm in the precordial leads). This clinical presentation is attributed to thickening of the left ventricular wall originating due to interstitial amyloid deposits, and not cardiomyocyte hypertrophy. However, this finding ensues at a later stage during the course of the disease. Indeed, only ~50% of patients with AL-CA and 20–40% of patients with ATTR-CA meet the low-voltage criteria [21,26,27]. Thus, absence of a low QRS voltage is not an adequate rationale to exclude CA. Another typical ECG feature associated with CA is the presence of a pseudo-infarct pattern with Q waves in precordial or limb leads, which is often present in ~70% of CA patients.

### 4.2. Multimodal Imaging

Currently, there is no single imaging modality that can perform the detailed diagnosis as well as assessment of the morphologic/functional consequences of CA. Thus, the practical application of multimodal imaging is essential for the clinical evaluation of CA. Recently, radionuclide bone scintigraphy has been recognized as a noninvasive diagnostic tool for the detection of ATTR-CA in the absence of monoclonal protein. However, bone scintigraphy alone is not enough to evaluate the structure and function of CA. Thus, echocardiography remains an important imaging tool in the detection and management of CA as it serves to be useful in raising suspicion, differentiation of other diseases, and monitoring the treatment outcomes. Cardiovascular magnetic resonance (CMR) can further provide tissue characterization and high-resolution structural and functional assessment of CA. Thus, these multimodal imaging techniques should be employed in a complementary manner to increase the diagnostic yield for CA [2,28,29,30,31,32].

#### 4.2.1. Echocardiography

**Figure 2:**
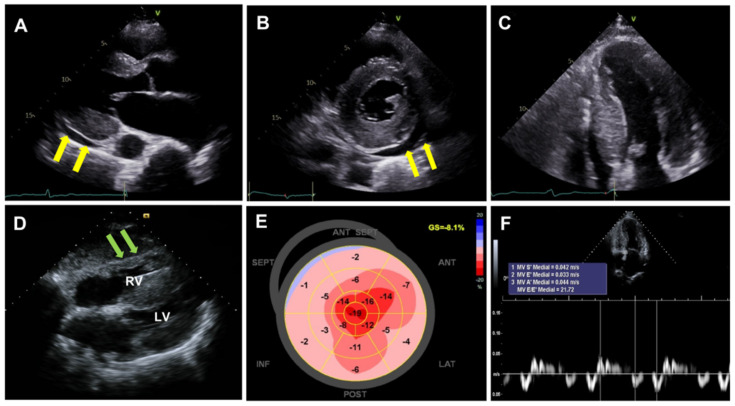
Echocardiographic findings of cardiac amyloidosis. (**A**–**C**): Thickened left ventricular wall (≥12 mm) with a granular sparkling appearance of myocardial texture and a small amount of pericardial effusion (yellow arrow), (**D**): right ventricular free wall hypertrophy (green arrow), (**E**): apical sparing of longitudinal strain (bull’s eye or cherry on top pattern), (**F**): deteriorated diastolic function.

Echocardiography is an advantageous and widely applicable diagnostic technique employed for the evaluation of patients with suspected heart failure symptoms [28,33,34]. Combination of increased left ventricular wall thickness (≥12 mm) on echocardiography and low to normal QRS voltage on ECG is the hallmark of CA. It is crucial to accurately measure left ventricular wall thickness using the parasternal long-axis view. Additional CA-associated features include small left ventricular cavity volume, biatrial enlargement, thickened valves and right ventricular free wall, restrictive diastolic pattern, and a small amount of pericardial effusion (Figure 2) [2,28]. Interestingly, pericardial or pleural effusion has been documented in more than half of CA patients [35], thus acting as a distinguishing feature from hypertrophic cardiomyopathy [34]. Potential mechanisms for pericardial or pleural effusion in CA include right ventricular failure and serosal infiltration of amyloid deposits. It is known that pericardial or pleural effusion alone is not specific to CA; it can also be found in many other inflammatory cardiac diseases, particularly in cases involving the right heart. However, pericardial effusions along with other echocardiographic peculiarities warrant clinical suspicion of CA [34]. A “granular sparkling” appearance of myocardial texture has been conventionally considered as a typical echocardiographic feature [34]. However, it has been confirmed to be a nonspecific finding [36,37] as it is also frequently manifested in other conditions, such as end-stage renal disease. Moreover, the harmonic echocardiographic imaging technique could result in confounding findings [38].

A wide spectrum of diastolic dysfunctions, ranging from grade 1 impaired relaxation to grade 3 restrictive filling, can be detected by the severity of amyloid infiltration [2,28]. It has been documented that the left ventricular ejection fraction is usually preserved until the late phase of CA progression [27,39]. Despite the preserved ejection fraction, left ventricular performance is known to deteriorate from the earlier phases [39,40,41]. The early stages of CA are characterized by infiltration originating in the sub-endocardium, which mainly comprises longitudinal fibers [38]. Therefore, longitudinal indices of myocardial contraction (not the radial/circumferential ones) may serve as early markers of systolic dysfunction [38,39]. Particularly, regional longitudinal strain variation, referred to as “relative apical sparing” might be helpful in distinguishing CA from other causes of left ventricular hypertrophy [42]. In CA, a longitudinal strain typically suggests an impaired basal/mid-segmental strain but preserved apical longitudinal strain, which results in “bull’s eye or cherry on top pattern” when the segmental strain is plotted (Figure 2). Several metrics have been suggested to objectively quantify the degree of relative apical sparing. Phelan et al. proposed the relative apical sparing index as follows: average apical longitudinal strain/average basal and mid-longitudinal strain. In their study, the ratio of 1.0 showed appropriate sensitivity and specificity in differentiating CA from other causes of hypertrophy (hypertrophic cardiomyopathy and aortic stenosis) [42]. Liu et al. calculated the septal apical longitudinal strain/septal basal longitudinal strain, and the cutoff value of >2.1 showed an optimum diagnostic performance in differentiating other etiologies of hypertrophy [43]. The suggested mechanisms include (1) less amyloid deposition at the apex than at the base, (2) greater diversity of myocyte and matric orientation at the apex as compared with the base, and (3) greater tendency toward apoptosis and remodeling in the basal segment related to turbulent flow in the left ventricular outflow tract and higher parietal stress [44,45].

Individual classic/novel echocardiographic parameters, or combinations of these parameters, have been extensively studied. Relative wall thickness (RWT) has been suggested as an important echocardiographic measure for raising the suspicion of ATTR-CA [4,46,47]. Remarkably, most echocardiographic characteristics may not be prominent in the early stages of disease progression and lack specificity to distinguish CA from other restrictive or hypertrophic cardiomyopathy [37]. Hence, better quality echocardiographic images and meticulous interpretation of the results hold utmost importance, as it is the most preferentially employed screening modality in CA detection.

#### 4.2.2. Cardiac Magnetic Resonance

Cardiac magnetic resonance (CMR) serves an exclusive role in the diagnosis of CA in two ways: (1) it can provide high-resolution structural and functional data, and (2) more importantly, it can provide tissue characterization information. As a result, CMR offers clinical benefit in the diagnosis of CA because of its ability to differentiate CA from other diseases that lead to increased wall thickness (e.g., hypertrophic cardiomyopathy, Fabry disease). A comprehensive CMR evaluation of CA involves structural and functional assessment of the heart using cine imaging, evaluation of native T1 signal, assessment of late gadolinium enhancement (LGE), and extracellular volume (ECV) measurement.

Notably, CMR is capable of accurately characterizing the myocardial tissue. Specifically, the intrinsic magnetic signals from the myocardium, which are measured as the magnetic resonance parameters T1, T2, and T2*, can serve to be useful in distinguishing a normal myocardium from an abnormal one, without the use of gadolinium-based contrast agents. However, these intrinsic properties can be further highlighted by the administration of gadolinium-based contrast agents, through the application of the LGE technique and ECV calculation [2,48]. Gadolinium is an exclusively extracellular agent that cannot cross the cell membranes of intact cardiomyocytes. In CA, the extracellular space expands due to increased amyloid infiltration, which enhances the gadolinium concentration in the myocardium, thereby showing hyperenhancement [2].

**Figure 3:**
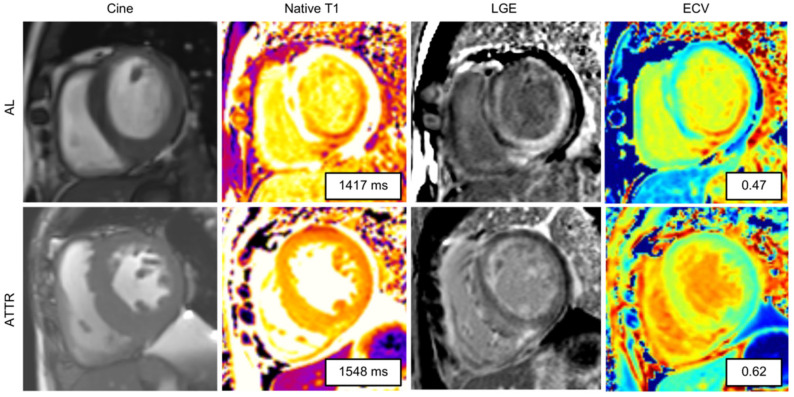
Cardiac magnetic resonance (CMR) findings of cardiac amyloidosis. Figure 3 shows end-diastolic cine stills, native T1 maps, late gadolinium enhancement (LGE) images, and extracellular volume (ECV) maps in patients with AL (top row) and ATTR (bottom row) CA. Note the thickened ventricular wall in cine imaging. In cardiac amyloidosis, native T1 and ECV were elevated. LGE imaging demonstrated diffuse subendocardial to transmural hyper-enhancement.

The typical LGE pattern in CA is a global subendocardial myocardial hyperenhancement in a noncoronary artery territory (Figure 3). It was hypothesized that the LGE pattern progresses from no LGE, to subendocardial LGE, and to transmural LGE in the later stages of the disease [48,49,50]. Although many authors have referred to a “typical LGE pattern” in CA, current research suggests a more variable LGE pattern than those previously reported [48,51]. Another important finding in CA is abnormal myocardial and blood-pool gadolinium kinetics. The blood pool has a characteristic dark appearance in CA, probably because the gadolinium moves out faster from the blood pool and distributes into the total body amyloid load [49].

In diffuse diseases such as CA, nulling, i.e., rendering, the normal myocardium dark is difficult with traditional LGE imaging based on the inversion recovery method, because the operator chooses the null inversion time in accordance with what is considered to be a normal myocardium. In addition, abnormal contrast agent kinetics makes the traditional LGE imaging even more challenging. The myocardium reaches the null points earlier than the blood pool due to the global T1 shortening in CA. Conversely, in a normal heart, the blood pool becomes black first, and then the myocardium reaches the null point (black) [52]. Furthermore, the accuracy of a chosen null inversion time depends on clearance of the contrast agent, and patient tolerance to additional breath-hold acquisitions [2]. With the phase-sensitive inversion recovery (PSIR) imaging, the tissue with the least contrast will always be nulled, enabling PSIR reconstruction images to be more reliable and operator-independent [50]. LGE has been reported to be a strong prognosticator of mortality in CA patients as no LGE exhibits the most favorable prognosis, while transmural LGE demonstrates the worst prognosis [50].

However, LGE imaging is not suitable for certain patients with CA who often have renal impairment and are contraindicated to receive contrast agents. Here, the native T1 values could be more useful. T1 mapping represents a pixel-wise illustration of myocardial T1 relaxation times, which is a property intrinsic to each tissue, where very high T1 values are considered to be characteristic of CA. Native T1 (noncontrast T1) mapping has been reported to be increased in the absence of LGE, suggesting its role as an early disease marker [53]. However, native T1 is a composite signal from the extracellular and intracellular space. Thus, administration of contrast combined with ECV measurement enables to isolate the signal from the extracellular space [52,54]. ECV assessment has been documented to correlate well with technetium-99m (^99m^Tc)-3,3-diphosphono-1,2-propanodicarboxylicacid (DPD) bone scintigraphy [55]. It has been reported that both native T1 and ECV correlate with ATTR-CA mortality; however, ECV was the only independent prognostic parameter [55]. Therefore, it is better to acquire both native T1 and ECV by contrast administration (Figure 3), whenever possible [54,56].

ATTR-CA is known to have more asymmetrical septal hypertrophy [57] and more extensive and transmural patterns of LGE than AL type [58]. It has been also reported that ECV is more elevated in ATTR-CA than AL-CA, reflecting more amyloid burden, while native T1 and T2 tend to be lower in ATTR-CA than AL-CA, probably due to myocardial edema induced by the toxic effect of AL amyloid fibrils [59]. However, it is important to note that CMR cannot definitively differentiate between AL-CA and ATTR-CA.

Although CMR is considered to be a vital noninvasive imaging modality in patients with suspected or confirmed CA, its results should be interpreted with biomarker, electrocardiographic, echocardiographic, and other imaging findings. Further, local availability of the technique and expertise should also be taken into consideration [52].

#### 4.2.3. Bone Scintigraphy and Single-Photon Emission Computed Tomography

Bone scintigraphy is now recognized as a confirmatory diagnostic tool for ATTR-CA in the absence of an invasive cardiac biopsy. Bone scintigraphy is widely available, without the need for restrictions of diet or medications, or prosthesis-associated contraindications. In the last decade, three ^99m^Tc-labeled bone-seeking radiotracers (^99m^Tc-pyrophosphate (PYP), ^99m^Tc-hydroxydiphosphonate (HMDP), ^99m^Tc-DPD) have been introduced for the diagnosis of ATTR-CA, with different radiotracers being used in different countries (^99m^Tc-PYP in the US, ^99m^Tc-HMDP in France, and ^99m^Tc-DPD in other countries, including the UK). Although the diagnostic yields of these radiotracers are generally considered similar, very few studies have directly compared their diagnostic yields specifically [2,29,60]. In a multicenter study of 374 patients each using three different bone-seeking radiotracers, ^99m^Tc-PYP and ^99m^Tc-DPD showed higher sensitivity but lower specificity than ^99m^Tc-HMDP for detecting CA (sensitivity of 89%, 89%, and 78% and specificity of 89%, 78%, and 100% for ^99m^Tc-PYP, ^99m^Tc-DPD, and ^99m^Tc-HMDP, respectively). The ^99m^Tc-DPD had a slightly higher sensitivity and specificity than ^99m^Tc-PYP in differentiating ATTR-CA from AL-CA [61,62]. Not all bone-seeking radiotracers are suitable for diagnosis of ATTR-CA. For example, ^99m^Tc-methylene diphosphonate, one of the most frequently used radiotracers for evaluation of bony abnormality, is not recommended for diagnosis of ATTR-CA due to its low sensitivity. The mechanism of differential myocardial uptake of these radiotracers in ATTR-CA and AL-CA is not well understood but is thought to be related to the differential presence of microcalcifications in the heart [63].

**Figure 4:**
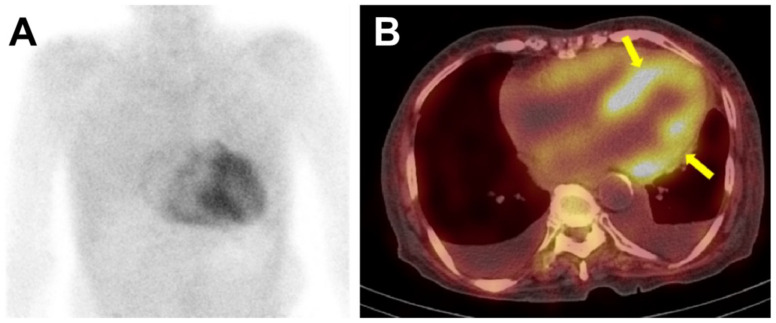
The ^99m^Tc-DPD bone scintigraphy and single-photon emission computed tomography (SPECT) of a patient with ATTR-cardiac amyloidosis. In Figure 4, the anterior planar image (panel **A**) showed intense, heterogeneous cardiac uptake greater than rib uptake in intensity, considered as Perugini grade 3. No abnormal radiotracer uptake was seen in other organs such as the liver or kidney. SPECT images (panel **B**) were acquired immediately after the planar imaging, and the radiotracer uptake could be localized to the myocardium (arrows), and not in the blood pool. Bilateral pleural effusion was also noted.

Bone scintigraphy involves the intravenous administration of 370 to 925 MBq of ^99m^Tc-labeled bone-seeking radiotracer followed by planar and single-photon emission computed tomography (SPECT) imaging after 1–3 h. An assessment of myocardial radiotracer uptake is the mainstay in the diagnosis of ATTR-CA. The intensity of myocardial radiotracer uptake can be analyzed either by visual grading or by quantifying radiotracer uptake using the heart-to-contralateral lung ratio (H/CL ratio). The Perugini grading system is based on a visual analysis of cardiac uptake at 2–3 h delayed planar image where (1) grade 0 indicates no cardiac uptake, (2) grade 1 suggests mild cardiac uptake less than that in rib, (3) grade 2 suggests moderate cardiac uptake equal to that in rib, while (4) grade 3 indicates intense cardiac uptake greater than that in rib. Bone scintigraphy with a Perugini grade of 2 or 3 has been reported to demonstrate a high sensitivity of about 99% for ATTR-CA, but a lower specificity ranging from 82–86%, given that a grade of 1–2 can be also observed in AL-CA. However, if monoclonal gammopathy was excluded via the urine/serum test, the specificity of the test was demonstrated to be increased to almost 100% [2]. Another quantification method using the H/CL ratio is evaluated in a 1 h image after ^99m^Tc-PYP administration [64,65]. Here, an H/CL ratio ≥ 1.5 is highly suggestive for ATTR-CA, and therefore can be used in the diagnosis of ATTR-CA. In addition to its role in diagnosis, an H/CL ratio ≥ 1.6 can be used as a worse prognostic marker [65]. An important caveat in assessment of myocardial radiotracer uptake is that a planar image alone has limitation in distinguishing between true myocardial uptake and blood pool uptake. Therefore, additional SPECT imaging is necessary to achieve more accurate localization of radiotracer uptake (Figure 4) [29].

In the current guidelines, in the context of the “red flag” signs and typical echocardiography/CMR findings for CA, (1) the presence of ≥grade 2 uptake or H/CL ratio ≥ 1.5 in bone scintigraphy confirmed by SPECT, and (2) absence of monoclonal gammopathy by immunofixation electrophoresis and serum-free light chain assay, are accepted as a definitive diagnosis of ATTR-CA in the absence of tissue biopsy [66]. Conversely, in case of any uptake on bone scintigraphy with positivity for monoclonal gammopathy, biopsy is essential to identify AL-CA. Further, in cases of grade 1 uptake on bone scintigraphy without the evidence of monoclonal gammopathy, a cardiac biopsy is recommended if CA is highly suggestive.

#### 4.2.4. Positron Emission Tomography

Positron emission tomography (PET) is an emerging diagnostic tool for diagnosing CA. Several PET tracers, such as ^18^F-florbetapir, ^18^F-flutemetamol, ^18^F-florbetaben, and ^11^C-Pittsburgh B (^11^C-PiB), have been invested for CA imaging [2,67,68,69]. These PET tracers are thioflavin-T analogs. Thioflavin-T is a histological dye similar to Congo red dye that shows increased fluorescence when it binds to the beta-pleated motif of the amyloid fibril. Although these PET amyloid tracers bind to any type of amyloid fibril, they seem to have a higher affinity for AL than for ATTR, which is opposite to the characteristic of bone-seeking radiotracers [67]. In a pilot study involving 19 subjects (14 CA patients and 5 controls), ^18^F-florbetapir retention was higher in CA patients than in control [69]. Similarly, Lee et al. reported the usefulness of ^11^C-PiB PET for detecting CA. Furthermore, they provided a quantitative assessment of amyloid burden (maximal myocardium-to-blood cavity ratio) in patients with and without chemotherapy, which implies the possibility of ^11^C-PiB PET as a monitoring tool for therapeutic response [69]. However, large-scale studies are required for practical application.

#### 4.2.5. Role of Multimodal Imaging in Follow-Up and Treatment Monitoring

To date, no studies have addressed the optimal follow-up scheme of multimodal imaging in patients with CA. Recently, the European Society of Cardiology (ESC) working group on myocardial and pericardial diseases proposed a follow-up scheme for CA patients. Specifically, the follow-up schedule differed in accordance with the type of CA with more frequent visits recommended in case of AL-CA, particularly during initial hematologic treatment [4]. In ATTR-CA patients, the recommended follow-up scheme involved 6-month visits with ECG and complete blood tests (including NT-proBNP and troponin) in addition to yearly echocardiography and 24 h Holter monitoring [4]. Some studies have reported the role of CMR in monitoring the treatment outcomes of patients [70,71]. Additional research is required regarding the choice of imaging modalities that are appropriate for tracing therapy-related changes.

### 4.3. Biomarker

The role of biomarkers in CA is mainly associated with risk stratification and monitoring the treatment responses. In AL-CA, quantification of serum-free light chain (FLC) and identification of monoclonal protein on immunofixation and electrophoresis (both serum and urine) have a high sensitivity (99%) for identifying AL-CA [61,72]. However, up to 5% of the elderly population aged ≥ 65 years has been reported to possess monoclonal gammopathy of undetermined significance (MGUS); thus, an abnormal kappa to lambda ratio alone is not specific for AL amyloidosis and biopsy is obligatory [64]. One important caveat when interpreting FLC concentration is that renal function should be brought under consideration, given that FLCs are filtered by the kidney. Consequently, a different normal reference range for FLCs should be adopted to diagnose AL-CA with chronic kidney disease. For instance, the normal kappa to lambda ratio is 0.26–1.65, but a higher range of 0.37–3.1 is considered normal in chronic kidney disease [4,73].

Among the various biomarkers, natriuretic peptides and troponin levels have been widely evaluated in patients with CA. Natriuretic peptides tend to be elevated in CA patients, out of proportion to the left ventricular systolic function. Perfetto et al. have reported that plasma NT-proBNP levels are higher in patients with AL-CA than in ATTR-CA [74]. The direct toxic effect of the circulating light chains on myocytes might be involved in this phenomenon [13,74,75,76]. Palladini et al. have previously reported the clinical usefulness of NT-proBNP as a sensitive marker for cardiac involvement as well as a prognostic marker in AL-CA [77]. Troponin (troponin T, troponin I) is a sensitive and specific marker of cardiac injury [78]. Elevated cardiac troponin levels in light chain amyloidosis are associated with poor survival [79].

#### 4.3.1. Biomarker Staging System for AL-CA

The first staging system for AL-CA was developed in 2004, termed as MAYO2004 with stages I to III. It was based on a combination of elevated serum NT-proBNP and cardiac troponin T or I at presentation. Current prognostic staging systems for AL-CA rely on the concentrations of FLCs, NT-proBNP, and troponin T, which also constitute the revised Mayo Clinic staging system (MAYO2012) [80]. In this staging system, the patients are assigned one point for each of the following indicators: FLC difference (kappa-lambda) ≥ 18 mg/dL, cardiac troponin T ≥ 0.025 ng/mL, and NT-proBNP ≥ 1800 pg/mL. Accordingly, stages I (0 points), II (1 points), III (2 points), and IV (3 points) were created with corresponding overall median survivals of 94, 40, 14, and 6 months, respectively. Alternatively, high-sensitivity cardiac troponin T can be used with a cutoff of ≥40 pg/mL [81]. Wechalekar et al. analyzed the prognostic value of the Mayo staging system in advanced AL-CA patients undergoing treatment. In their study, they only included patients with stage III disease (according to the MAYO2004 staging system). They demonstrated that high NT-proBNP (>8500 ng/L) and low systolic blood pressure (<100 mmHg) are independent markers of poor prognosis, which corresponds to the ultra-high-CA risk population (MAYO3b) [82].

#### 4.3.2. Biomarker Staging System for ATTR-CA

Currently, there are two prognostic scoring systems for the staging of ATTR-CA. The Mayo Clinic System in wtATTR-CA uses the same stages I to III as those described in the MAYO2004 for AL-CA. However, they use a different (higher) cutoff value for NT-proBNP (≥3000 pg/mL) and cardiac troponin T (≥0.05 ng/mL). Further, it defines the disease progression as stage I when both the values are below the cutoff point, stage II when either one of the values is above the cutoff point, and stage III when both the values are above the cutoff point. The overall median survival with this staging system has been reported to be 66, 40, and 20 months for stages I, II, and III, respectively [83]. Another new staging system has been developed, which can be applied in both wild and hereditary ATTR-CA. It is called the UK National Amyloidosis Center Staging System in wtATTR and hATTR. This system is based on NT-proBNP levels > 3000 pg/mL and eGFR levels < 45 mL/min. It defines stage I with NT-proBNP ≤ 3000 pg/mL and eGFR ≥ 45 mL/min, stage II with either NT-proBNP or eGFR above the cutoff levels, and stage III with both NT-proBNP and eGFR above the cutoff levels. The overall median survival for ATTR-CA patients classified with this staging system was determined to be 69, 47, and 24 months for stages I, II, and III, respectively [84].

Overall, currently available scoring systems have been constructed using biomarkers obtained “at presentation”, which do not reflect the changes during the follow-up. Hence, additional research is indispensable to elucidate the prognostic impact of changes in the scoring system during follow-up of CA patients [4].

#### 4.3.3. Biomarkers for Therapeutic Response in AL-CA

NT-proBNP is a useful biomarker for disease progression or response to therapy for AL-CA. According to the 2005 consensus guideline for the reporting of clinical trials in systemic AL, the heart response was defined as (1) NT-proBNP response > 30% and >300 ng/L decrease in patients with baseline NT-proBNP ≥ 650 ng/L or (2) NYHA functional class response ≥ 2 class decrease in subjects with baseline NYHA class 3 or 4. Conversely, the heart progression was defined as (1) NT-proBNP progression with >30% and >300 ng/L increase), (2) cardiac troponin progression with ≥33% increase, or (3) ejection fraction progression with ≥10% decrease [85,86]. However, caution is required before drawing final conclusions, as NT-proBNP or troponin levels can also be altered by renal excretion independent of the state of AL. It has also been shown that changes of 2 mm reduction in septal wall thickness by echocardiography in response to therapy are not associated with a survival benefit [86].

#### 4.3.4. Biomarkers for Therapeutic Response in ATTR-CA

NT-proBNP and troponin have been widely explored as ATTR-CA biomarkers for therapeutic response, albeit with inconsistent results [84,85,86]. With the availability of novel treatments, the role of biomarkers for therapeutic response or disease progression in ATTR-CA has emerged as an area of further investigation [3].

#### 4.3.5. Other Novel Biomarkers

Although troponins and natriuretic peptides have been widely evaluated, they usually represent late-phase pathophysiology of CA. Recently, novel biomarkers that can detect early- or mid-phase pathophysiology of CA have been investigated. Soluble suppression of tumorigenicity 2 (sST2), which reflects cardiac fibrosis, has been reported to be associated with adverse outcomes in AL-CA. A value of 30 ng/mL was considered as cutoff in their study [87]. Hepatocyte growth factor (HGF) has been also suggested as a novel biomarker of AL-CA. Swiger et al. reported that HGF might help discriminate AL-CA from other cardiomyopathies. Interestingly, HGF was found to be elevated only in the patients with systemic AL with cardiac involvement [88]. Osteopontin and osteoprotegerin, which reflect bone remodeling, have been studied in AL-CA [89,90]. Increased levels of osteoprotegerin in AL-CA were correlated with NT-proBNP [89]. Kim et al. examined the prognostic value of novel biomarkers (sST2, growth differentiation factor [GDF] 15, and osteopontin) in AL patients. In their study, sST2 and GDF-15 showed appropriate prognostic value for overall survival and additive incremental value over conventional biomarkers (NT-proBNP and troponin T) [91]. Unfortunately, most novel biomarkers are not specific to CA and there is a lack of data for ATTR-CA. The development of additional novel biomarkers that can capture early phase CA (both AL- and ATTR-CA) will be helpful for early diagnosis and better treatment in patients with CA.

## 5. Conclusions

**Figure 5:**
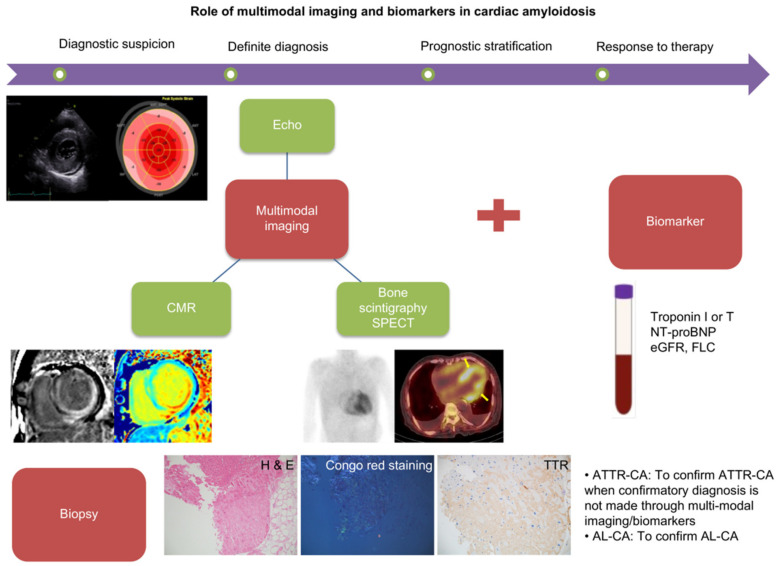
Role of multimodal imaging and biomarkers in cardiac amyloidosis.

Underrecognition and delayed diagnosis of CA might be overcome with the advancement in the multimodality imaging and biomarker assessment. Patients with CA who progress to advanced heart failure may not sufficiently benefit with existing CA therapies [88,89,90,91,92,93,94,95,96,97,98,99,100,101]. Therefore, early diagnosis of CA is essential so that effective therapies can be initiated early enough in the disease course. Multimodal imaging techniques such as echocardiography with strain analyses, CMR, and bone scintigraphy have emerged as tools of paramount significance in the diagnosis of CA. However, these techniques may be more effective when employed in conjunction with each other to increase the diagnostic accuracy. In addition, certain CA biomarkers, such as NT-proBNP and troponin, may serve to be helpful in risk stratification and the monitoring of treatment responses in CA patients (Figure 5).

Comprehensive and integrative evaluation using multimodal imaging, biomarkers, and biopsy is crucial in the diagnosis, risk stratification, and monitoring of response to therapy in cardiac amyloidosis. Please refer to the main text regarding the specific role of each imaging modality and biomarker.

## Data Availability

Not applicable.
